# Neobractatin and Trametinib Synergistically Induce Apoptosis and Gasdermin E‐Dependent Pyroptosis in Pancreatic Cancer Cells

**DOI:** 10.1002/mco2.70250

**Published:** 2025-07-01

**Authors:** Jiaqi Tan, Ziyi Bao, Kai Qin, Liujing Zhu, Changwu Zheng, Jiabin Jin, Li Zhang, Hongxi Xu

**Affiliations:** ^1^ School of Pharmacy Shanghai University of Traditional Chinese Medicine Shanghai China; ^2^ Department of General Surgery Pancreatic Disease Center Ruijin Hospital, Shanghai Jiao Tong University School of Medicine Shanghai China; ^3^ Research Institute of Pancreatic Diseases, Shanghai Jiao Tong University School of Medicine Shanghai China; ^4^ Shuguang Hospital, Shanghai University of Traditional Chinese Medicine Shanghai China

**Keywords:** AKT, gasdermin E, pancreatic ductal adenocarcinoma, pyroptosis, ROS

## Abstract

Mutations in mitogen‐activated protein kinase kinase (MEK) are prevalent in pancreatic ductal adenocarcinoma (PDAC), but many MEK inhibitors inadvertently activate protein kinase B (AKT). We propose a promising PDAC treatment strategy by combining the MEK inhibitor trametinib with neobractatin (NBT), a natural compound from *Garcinia bracteata*. Our results demonstrated that this combination significantly impeded cell growth by inducing gasdermin E (GSDME)‐mediated pyroptosis and apoptosis. GSDME, overexpressed in PDAC tissues and correlated with histological differentiation, underscores the role of pyroptosis in PDAC. RNA‐seq results indicated that the phosphoinositide 3‐kinase/protein kinase B (PI3K/AKT) pathway was the primary target of the combination treatment. Mechanistic studies revealed the combination effectively reduced both total and phosphorylated AKT levels, thereby inhibiting protein kinase B/IκB kinase (AKT/IKK) and protein kinase B/mammalian target of rapamycin (AKT/mTOR) signaling pathways. Additionally, the combination disrupted mTOR complex 2 (mTORC2), preventing the trametinib‐induced AKT activation. MicroRNA sequencing analysis indicated that the combination reduced AKT levels by upregulated miR‐149‐5p. Further research demonstrated that the combination increased intracellular reactive oxygen species (ROS), while N‐acetylcysteine (NAC, a ROS scavenger) reversed the cell growth inhibition and AKT suppression. In vivo, the combination significantly inhibited tumor growth by inducing pyroptosis and apoptosis, outperforming gemcitabine. Our findings provide novel insights into the potential of combining NBT and trametinib to induce pyroptosis and apoptosis through the ROS/AKT/GSDME axis, offering a theoretical basis for future PDAC treatment.

## Graphical abstract

Neobractatin, a natural compound derived from *Garcinia bracteata*, significantly inhibited the growth of PDAC cells when used in combination with trametinib. This combination therapy promoted cell death through GSDME‐mediated pyroptosis and apoptosis. Mechanistically, the combined treatment elevated levels of ROS, which in turn suppressed the AKT signaling pathway and its downstream targets, thereby enhancing the anti‐tumor efficacy against PDAC cells.

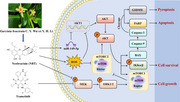

## 1 Introduction

Pancreatic ductal adenocarcinoma (PDAC) is a highly malignant gastrointestinal tumor and ranks as the seventh leading cause of cancer‐related deaths worldwide [1]. According to the latest data from the National Cancer Center, PDAC is the tenth most commonly diagnosed cancer and the sixth leading cause of cancer mortality in China [2]. PDAC is one of the tumors with the poorest prognosis among solid tumors. Most patients with PDAC present symptoms only in the late stages of the disease, often diagnosed with metastatic or locally advanced disease, with a small proportion eligible for surgical resection [3]. The median survival after treatment is approximately 10–12 months. The 5‐year survival rates for patients diagnosed with localized disease are 37%, whereas those with advanced disease have a lower rate of 3% [4]. Due to the limited number of patients eligible for surgical resection, chemotherapy has become an important therapeutic approach for treating PDAC. It can effectively improve the 5‐year overall survival (OS) rate of patients. Currently, the first‐line treatment options include FOLFIRINOX (a combination of fluorouracil, calcium folinate, irinotecan, and oxaliplatin) or gemcitabine combined with albumin‐bound paclitaxel [5].

The mitogen‐activated protein kinase (MAPK) pathway is integral to tumor cell proliferation and survival [6, 7]. Targeted therapies, such as trametinib, a selective, orally bioavailable MEK1/2 inhibitor, have shown promise in treating cancers by inhibiting key molecular targets within this pathway [6, 7]. Mutations in MEK, particularly downstream of oncogenic Kirsten rat sarcoma (KRAS) signaling, are critical in the development and progression of pancreatic cancer and contribute to resistance against both targeted therapies and conventional chemotherapy [8]. While trametinib has been United States Food and Drug Administration‐approved for B‐Raf proto‐oncogene (BRAF) V600E/K+ unresectable or metastatic melanoma, either alone or in combination with dabrafenib for melanoma and non‐small cell lung cancer, its efficacy can be undermined by survival feedback mechanisms, including AKT pathway activation and autophagy [9, 10, 11]. These feedback mechanisms limit the drug's therapeutic effectiveness, prompting interest in combination strategies to overcome resistance and enhance antitumor activity [12, 13, 14]. For instance, trametinib combined with hydroxychloroquine has shown potential clinical efficacy in cancer treatment [15, 16]. Additionally, natural compounds, known for their antitumor properties and low toxicity, are being explored as synergistic partners in combination therapies, offering a promising approach to improve treatment outcomes [17].

Neobractatin (NBT) is a natural compound derived from *Garcinia bracteata* C. Y. Wu ex Y. H. Li, which has shown antitumor activity against a range of cancer types, such as lung cancer, breast cancer and esophageal cancer [18, 19, 20, 21]. Our previous study has reported that NBT downregulated the phosphorylation of AKT [18]. In this study, we investigated the potential synergistic effects of combining NBT with trametinib. Our study demonstrated that the cotreatment synergistically inhibited the viability of PDAC cells by inducing both pyroptosis and apoptosis. The underlying mechanisms involved not only the reversal of the abnormal activation of AKT phosphorylation induced by trametinib but also the synergistic suppression of total AKT expression, driven by reactive oxygen species (ROS) accumulation and miR‐149‐5p. These findings elucidate the mechanisms underlying the antitumor effects of NBT when used in combination with trametinib and unveil a promising strategy for enhancing therapeutic outcomes through strategic drug combinations.

## 2 Results

### 2.1 NBT and Trametinib Synergistically Inhibited the Growth of PDAC Cells

**FIGURE 1:**
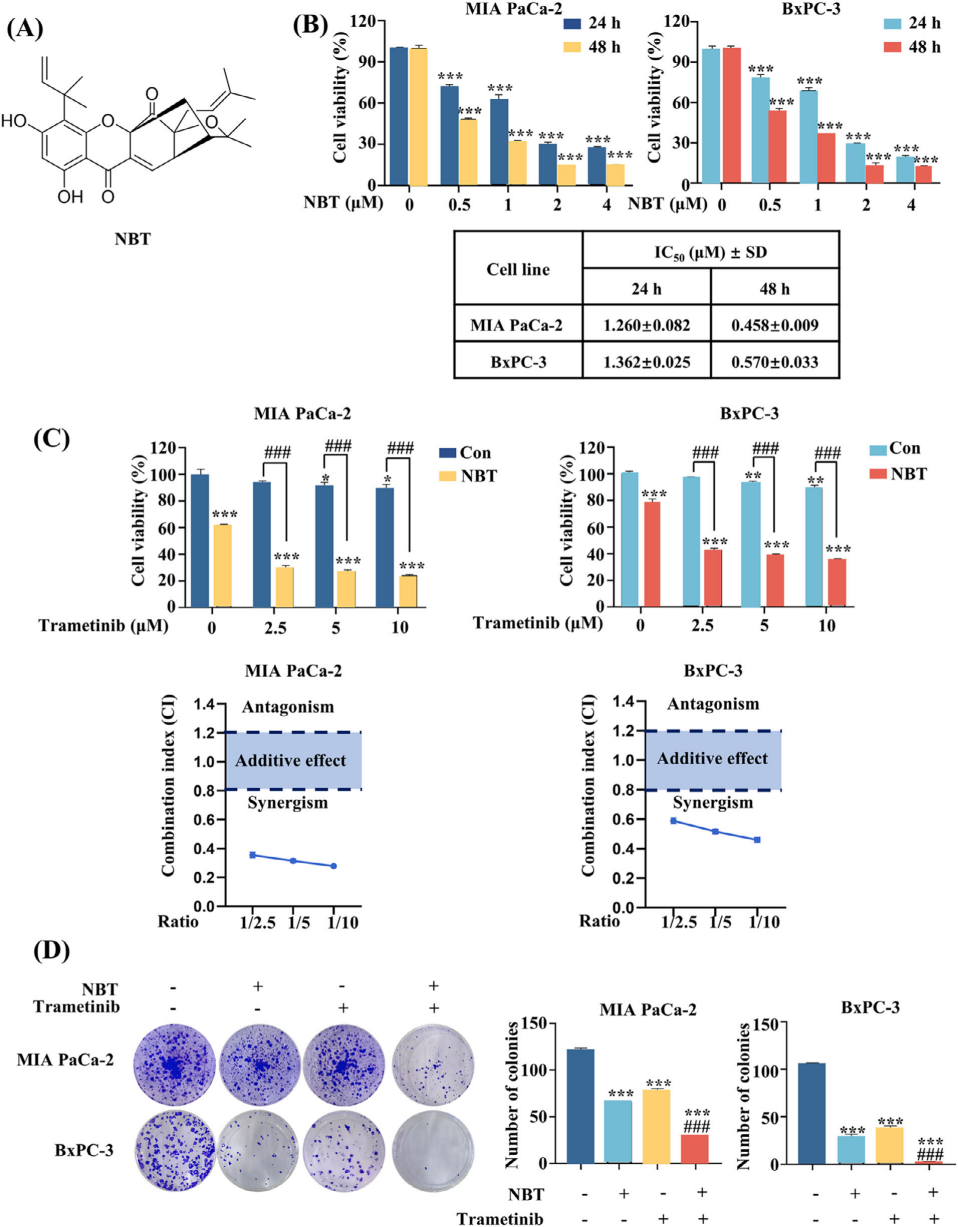
Synergistic antitumor effect of NBT in combination with trametinib. (A) The structure of NBT. (B) The IC_50_ of NBT in MIA PaCa‐2 and BxPC‐3 cells. (C) PDAC cells were treated with different concentrations of NBT and trametinib for 24 h and cell viability was detected by MTT assay. CI values were calculated by the corresponding formula. CI < 0.8 indicates synergism, CI from 0.8 to 1.2 indicates additive effect, and CI > 1.2 indicates antagonism. (D) The colony formation assay of PDAC cells was performed under mono or combination treatment of NBT (1 µM) and trametinib (5 µM) for 24 h. Then the cells were replaced new culture medium for another cultivation of 14 days. Data are presented as the means ± SD from three independent experiments. *p < 0.05, **p < 0.01, ***p < 0.001 versus control; ###*p* < 0.001 versus monotreatment.

To evaluate the cytotoxicity of NBT (Figure 1A) to PDAC cells, MTT assay was conducted to determine the IC_50_ value of NBT in MIA PaCa‐2 and BxPC‐3 cell lines. As depicted in Figure 1B, NBT inhibited cell proliferation in both dose‐ and time‐dependent manners, showing significant cytotoxicity in PDAC cells (IC_50_ < 1.5 µM). Consequently, the concentrations below the IC_50_ value, specifically 1 µM, were selected for the subsequent cotreatment. MTT assay showed that the combination of NBT and trametinib significantly inhibited the cell viability of PDAC cells (Figure 1C). The drug combination index (CI) was calculated using the Loewe Additivity model to assess the synergistic effect of the combination. Based on the CI value, the combination of NBT and trametinib exerted a strong synergistic effect on PDAC cells, with CI values <0.8. Furthermore, a colony formation assay was used to detect the potential effect on long‐term proliferation. The results showed that the combination of NBT and trametinib significantly impeded the colony formation of PDAC cells (Figure 1D). These findings indicated that the combination of NBT and trametinib synergistically inhibited the growth of PDAC cells.

### 2.2 NBT and Trametinib Synergistically Induced GSDME‐Dependent Pyroptosis and Apoptosis

**FIGURE 2:**
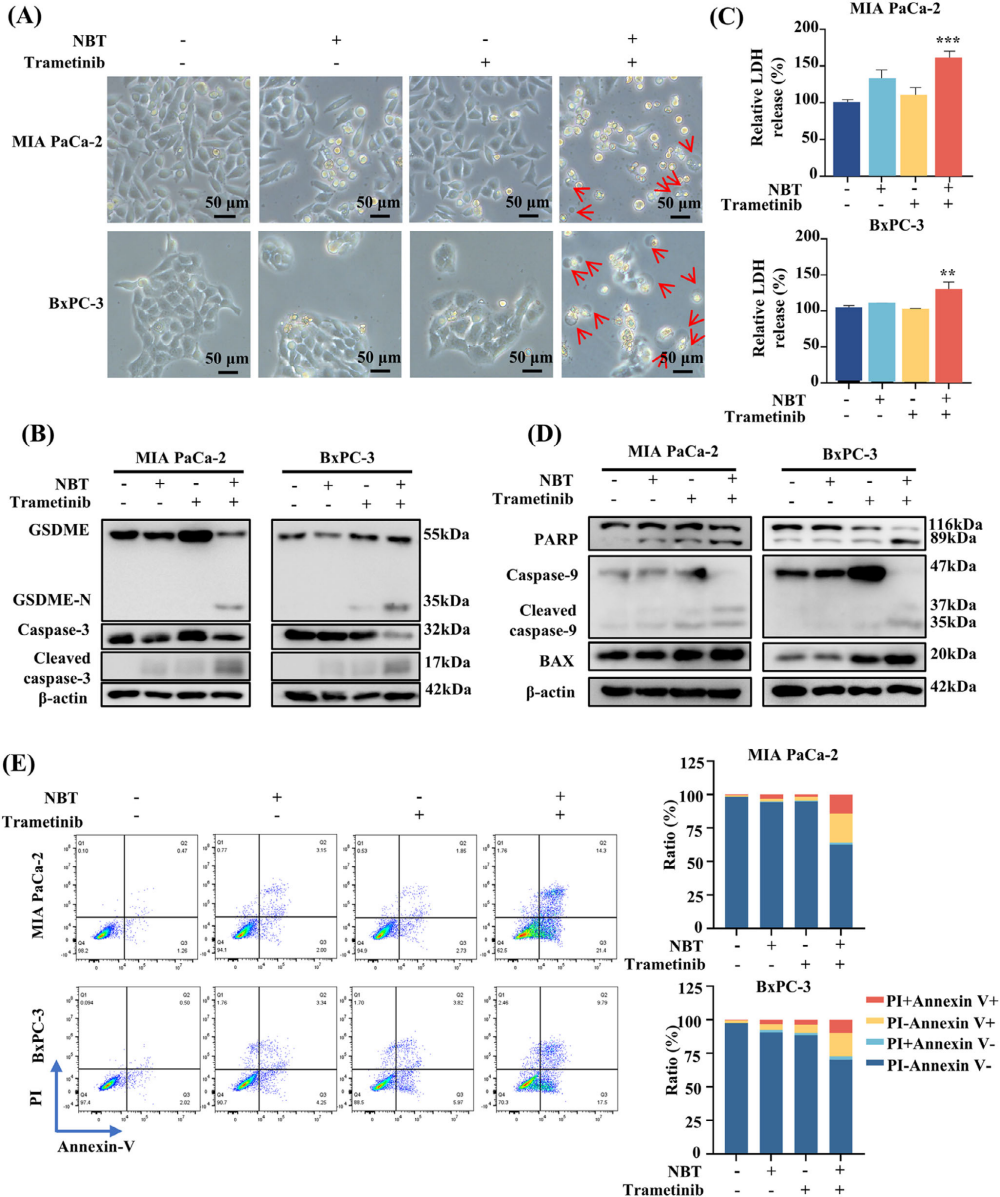
The combination of NBT and trametinib induced pyroptosis and apoptosis in PDAC cells. PDAC cells were treated with NBT (1 µM), trametinib (5 µM), or their combination for 24 h. (A) Bright‐field images were taken to observe cellular morphology (large bubbles are indicated by red arrows). (B) Western blot analysis of GSDME cleavage, with β‐actin as the control. (C) LDH release levels in PDAC cells after treatment. (D) Apoptosis‐related proteins (PARP, caspase‐9, BAX) were evaluated by Western blot, using β‐actin as the control. (E) Flow cytometry assessed apoptosis and pyroptosis using Annexin V and PI staining, with columns representing the percentage of stained cells. Data are presented as the means ± SD from three independent experiments. **p < 0.01, ***p < 0.001.

Morphologically, after the cotreatment of NBT and trametinib, PDAC cells exhibited swelling and distinct bubble‐like protrusions from the membrane, which are characteristic of pyroptotic cells (Figure 2A) [22]. Transmission electron microscope (TEM) showed multiple pores formed in the membranes of MIA PaCa‐2 cells after the cotreatment (Figure S1). Consequently, we examined the expressions of proteins related to pyroptosis. The combination prominently elevated the protein level of GSDME‐N, concomitant with the activation of caspase‐3 (Figure 2B). The cotreatment led to a notable increase in Lactate Dehydrogenase (LDH) release compared with the monotherapy, indicating the disruption of cell membrane integrity (Figure 2C). Since the integrity of the cell membrane could also be damaged during necroptosis, the necroptosis inhibitor GSK872 was employed to distinguish between pyroptosis and necroptosis. The results indicated that GSK872 did not influence the LDH release or cell death, supporting the occurrence of pyroptosis rather than necroptosis (Figure S2). Collectively, these evidences corroborated that NBT and trametinib synergistically induced GSDME‐dependent pyroptosis in PDAC cells.

Since caspase‐3 serves as both the key executor of GSDME in pyroptosis and a central effector in apoptotic processes, the effects of the combination on apoptosis were determined. Western blot results demonstrated the cotreatment activated Bcl‐2‐associated X protein (BAX) and caspase‐9 and enhanced the cleavage of poly(ADP‐ribose) polymerase (PARP), which are indicative of increased apoptosis (Figure 2D). Flow cytometry analysis further confirmed that the proportion of apoptotic (Annexin V^+^PI^−^) and pyroptotic cells (Annexin V^+^PI^+^) in response to the combination treatment compared with the monotherapy (Figure 2E).

### 2.3 GSDME is Overexpressed in PDAC Tissues

**FIGURE 3:**
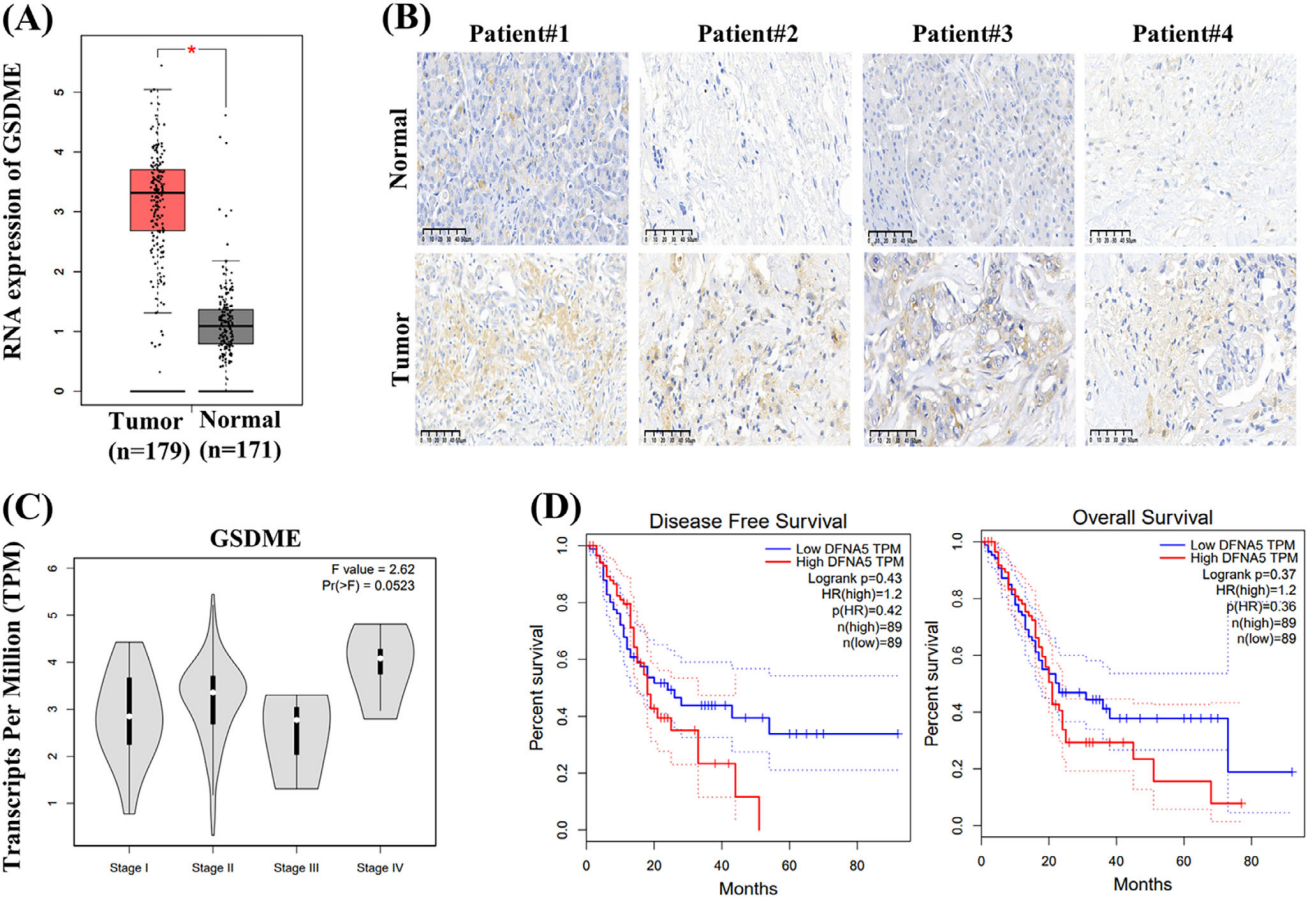
GSDME is highly expressed in PDAC tissues and associated with poor prognosis. (A) DNA copy number of GSDME in PDAC was analyzed using GEPIA database. (B) IHC staining of GSDME in PDAC tissues and adjacent normal tissues from patients. (C) Expression of GSDME in different clinical stages of PDAC patients. (D) The correlation between GSDME expression and disease‐free survival and overall survival of PDAC patients.

The expression of GSDME is of value in cancer progression and key to the efficacy of GSDME‐dependent pyroptosis [23, 24]. To investigate the clinical significance of GSDME in PDAC, we performed an in‐depth analysis through a large‐scale dataset analysis by using Gene Expression Profiling Integrative Analysis (GEPIA). The results revealed a significant upregulation of GSDME expression in PDAC tissues compared with normal tissues (Figure 3A). Consistent with these data, immunohistochemistry (IHC) analysis of tumor and adjacent normal tissues from surgical specimens further supported the high expression of GSDME in PDAC tissues (Figure 3B). Additionally, a positive correlation was observed between advanced clinical stages and increased GSDME expression levels (Figure 3C). Kaplan–Meier survival analysis suggested that high GSDME expression may be linked to shorter OS and disease‐free survival (DFS) in PDAC patients (Figure 3D).

### 2.4 Expression of GSDME Mediated the Switch Between Pyroptosis and Apoptosis with the Involvement of Caspase‐3

**FIGURE 4:**
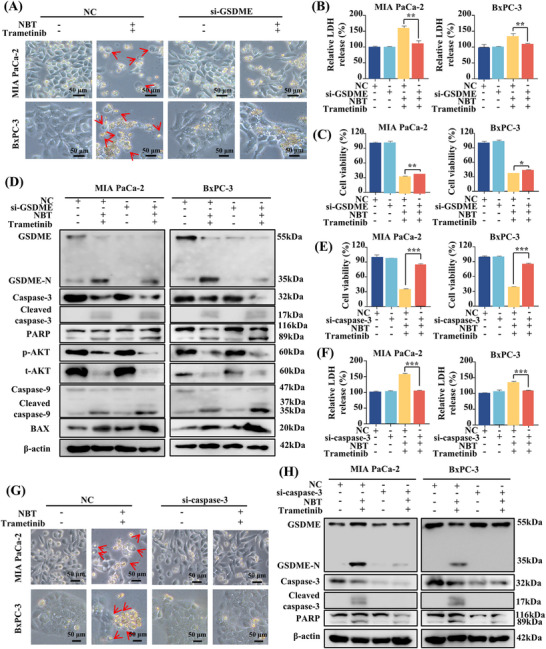
GSDME and caspase‐3 were involved in pyroptosis and apoptosis induced by the combination of NBT and trametinib. (A) Bright‐field images of PDAC cells after GSDME knockdown by siRNA (large bubbles marked by red arrows). (B) LDH release and (C) cell viability were measured. (D) Western blot analysis of GSDME, caspase‐9, caspase‐3, PARP, BAX, p‐AKT, and t‐AKT. (E) Cell viability and (F) LDH release after caspase‐3 knockdown. (G) Bright‐field images of PDAC cells after treatment. (H) Western blot analysis of GSDME, caspase‐3, and PARP. β‐actin was utilized as an internal control. Data are presented as the means ± SD from three independent experiments. *p < 0.05, **p < 0.01, ***p < 0.001.

It has been confirmed that GSDME‐dependent pyroptosis was engaged in cell death induced by the combination of NBT and trametinib. To further investigate the specific role of GSDME, we conducted siRNA‐mediated knockdown experiments. Notably, after knocking down GSDME, the characteristic morphological features of pyroptosis disappeared, and there was a significant reduction in LDH release from PDAC cells (Figure 4A,B). However, silencing GSDME only partially restored cell viability, implying the involvement of other modes of cell death (Figure 4C). Western blot analysis revealed that GSDME knockdown resulted in a substantial decrease in GSDME cleavage, indicating the blockage of pyroptosis (Figure 4D). Additionally, in the absence of GSDME, the cotreatment led to a more significant PARP cleavage and activation of BAX and apoptotic caspase‐9/3 (Figure 4D). These results suggested that silencing of GSDME facilitated the transition from pyroptosis to apoptosis.

Given that both GSDME‐mediated pyroptosis and apoptosis are regulated by activated caspase‐3, the role of caspase‐3 was also investigated [25]. MTT assay results demonstrated that the knockdown of caspase‐3 completely reversed the cell death and LDH release induced by the combination of NBT and trametinib (Figure 4E,F). Morphologically, the typical features of pyroptosis and even cell death were no longer observed in the absence of caspase‐3 (Figure 4G). Meanwhile, caspase‐3 knockdown effectively prevented the cleavage of both GSDME and PARP, suggesting the pivotal role of caspase‐3 in the induction of pyroptosis and apoptosis by the cotreatment with NBT and trametinib (Figure 4H).

### 2.5 The Combination of NBT and Trametinib Primarily Affected AKT Signaling Pathway

**FIGURE 5:**
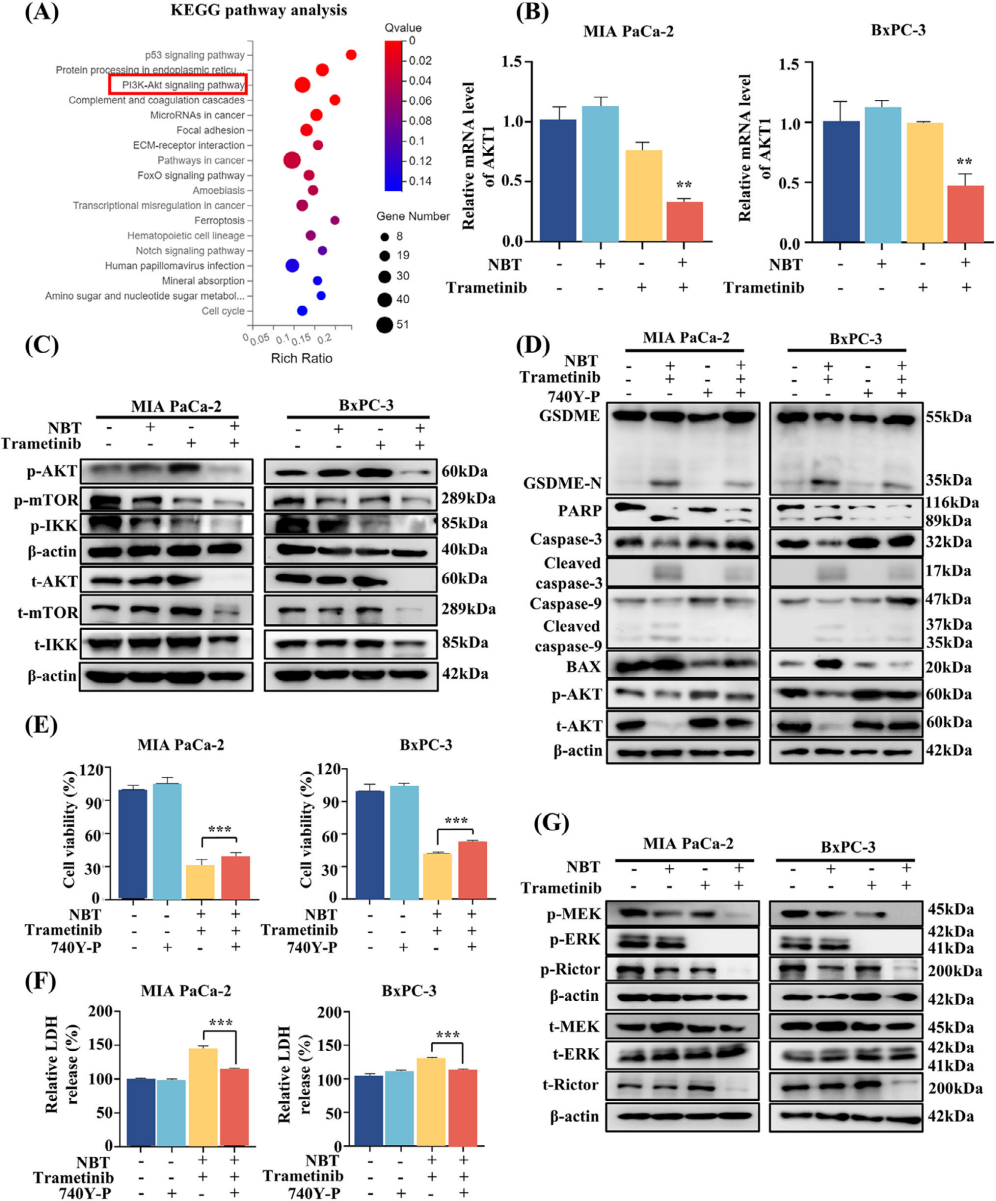
AKT signaling pathway played a vital role in the antitumor effects of the combination of NBT and trametinib. (A) MIA PaCa‐2 cells were treated with NBT (1 µM), trametinib (5 µM), or their combination for 12 h and subjected to RNA‐seq. KEGG pathway analysis identified differentially expressed genes between the control and combination groups. (B) mRNA levels of AKT1 in PDAC cells after treatment, with 18S as the control. (C) Western blot of p‐AKT, p‐mTOR, p‐IKKα/β, and their respective total proteins. (D) PDAC cells were pretreated with 740Y‐P (20 µg/mL) for 2 h before NBT and trametinib treatment. The protein levels of GSDME, PARP, caspase‐9, caspase‐3, BAX, p‐AKT, and total AKT were detected by Western blot. β‐actin was utilized as an internal control. (E) Cell viability and (F) LDH release levels were determined. (G) Western blot analysis of p‐MEK, p‐ERK1/2, and p‐Rictor and their total proteins. β‐actin was utilized as a control. Data are presented as the means ± SD from three independent experiments. **p < 0.01, ***p < 0.001.

To elucidate the underlying mechanism regulated by the combination of NBT and trametinib, RNA‐seq analysis was conducted. The differentially expressed genes were subjected to Kyoto Encyclopedia of Genes and Genomes (KEGG) enrichment analysis, which revealed a significant enrichment of the PI3K/AKT signaling pathway (Figures 5A and S3A). AKT has been previously confirmed to be highly expressed in most cancer tissues, including PDAC, when compared with normal tissues [26]. High expression of AKT1 mRNA was also observed in tumor tissue specimens (Figure S3B), although no statistical difference was detected between the adjacent and cancerous tissues, possibly due to the relatively small sample size in the analysis. The results of RNA‐seq analysis were confirmed in our study. As shown in Figure 5B, the cotreatment of NBT and trametinib significantly suppressed the mRNA level of AKT1. Western blot analysis further demonstrated that the cotreatment reduced both phosphorylation and total level of AKT, which was activated by trametinib alone (Figure 5C). Our study further proved that combination inhibited AKT activation at the transcriptional level rather than by altering protein degradation (Figure S4). The resulting downregulation of AKT further inhibited both total and phosphorylation levels of the downstream proteins, including mTOR and IKKα/β. Treatment with PI3K/AKT‐specific activator (740Y‐P) attenuated the cotreatment‐induced cleavage of GSDME and PARP and mitigated the activation of BAX and caspase‐9/3 (Figure 5D). MTT assay and LDH release assay also demonstrated that 740Y‐P partially inhibited cell death and reduced LDH release induced by the combination of NBT and trametinib (Figure 5E,F). These findings supported the crucial regulatory role of AKT in promoting pyroptosis and apoptosis in response to the combination treatment.

Furthermore, Western blot analysis revealed that the combination of NBT and trametinib significantly decreased the phosphorylation levels of MEK and ERK1/2 compared with trametinib alone (Figure 5G), indicating enhanced inhibition of the MAPK pathway. Previous studies have reported the crosstalk between MAPK and AKT signaling pathways mediated by mTORC1/2 complexes [27, 28]. Our results showed that the combination of NBT and trametinib significantly inhibited both phosphorylation and total expressions of Rictor, thereby reversing trametinib‐induced AKT activation.

### 2.6 ROS‐Mediated AKT Signaling Pathways Regulated Pyroptosis and Apoptosis

**FIGURE 6:**
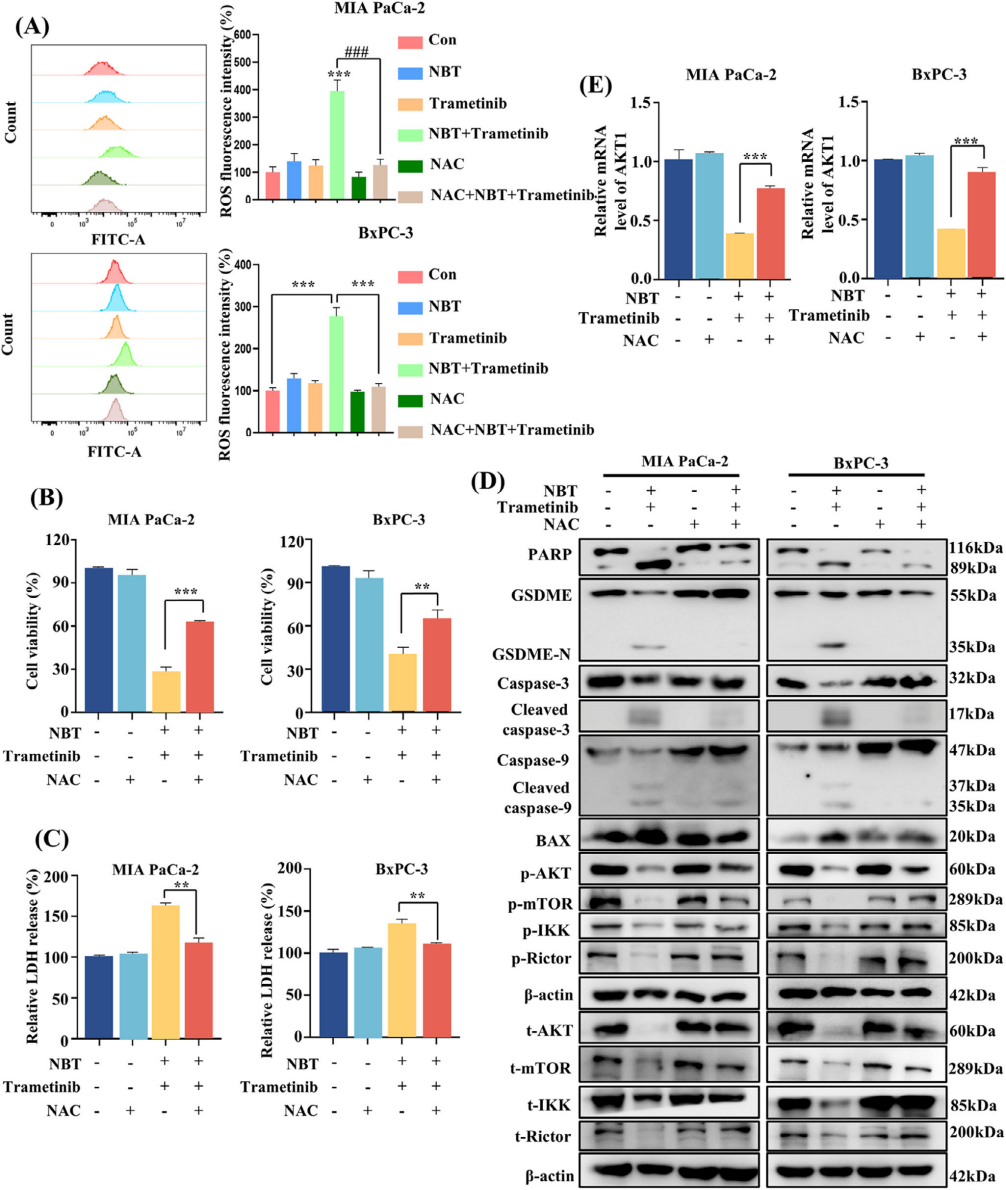
ROS mediated AKT signaling pathway to induce pyroptosis and apoptosis. (A) Flow cytometry was performed to measure intracellular ROS level by DCFH‐DA probe. (B) The cell viability and (C) LDH release levels were examined after combination treatment with or without NAC. (D) The protein levels related to apoptosis and pyroptosis, as well as AKT signaling pathway were detected by Western blot. β‐actin was utilized as an internal control. (E) The mRNA levels of AKT1 were detected. 18S was utilized as an internal control. Data are presented as the means ± SD from three independent experiments. **p < 0.01, ***p < 0.001.

Increased intracellular ROS levels not only cause DNA damage and apoptosis, but also participate in the regulation of pyroptosis [29, 30, 31]. The results of flow cytometry revealed that the cotreatment induced a significant increase in intracellular ROS levels (Figure 6A). Pretreatment with ROS scavenger N‐acetylcysteine (NAC) effectively attenuated the ROS levels induced by the cotreatment, leading to the restoration of cell viability and LDH release levels (Figure 6B,C). Moreover, Western blot results confirmed that the cleavage of GSDME and PARP, as well as the protein levels of BAX and caspase‐9/3 were effectively inhibited in the presence of NAC. Additionally, the downstream of AKT pathways, including mTOR and IKKα/β proteins, and upstream Rictor proteins were reactivated by NAC (Figure 6D). Simultaneously, the inhibitory effect on AKT at both the mRNA and protein levels was reversed upon NAC treatment (Figure 6E). These results strongly suggested that the combination of NBT and trametinib may induce pyroptosis and apoptosis through the regulation of ROS‐mediated AKT signaling pathways.

### 2.7 The Combination of NBT and Trametinib Upregulated miR‐149‐5p Expression Targeting AKT

**FIGURE 7:**
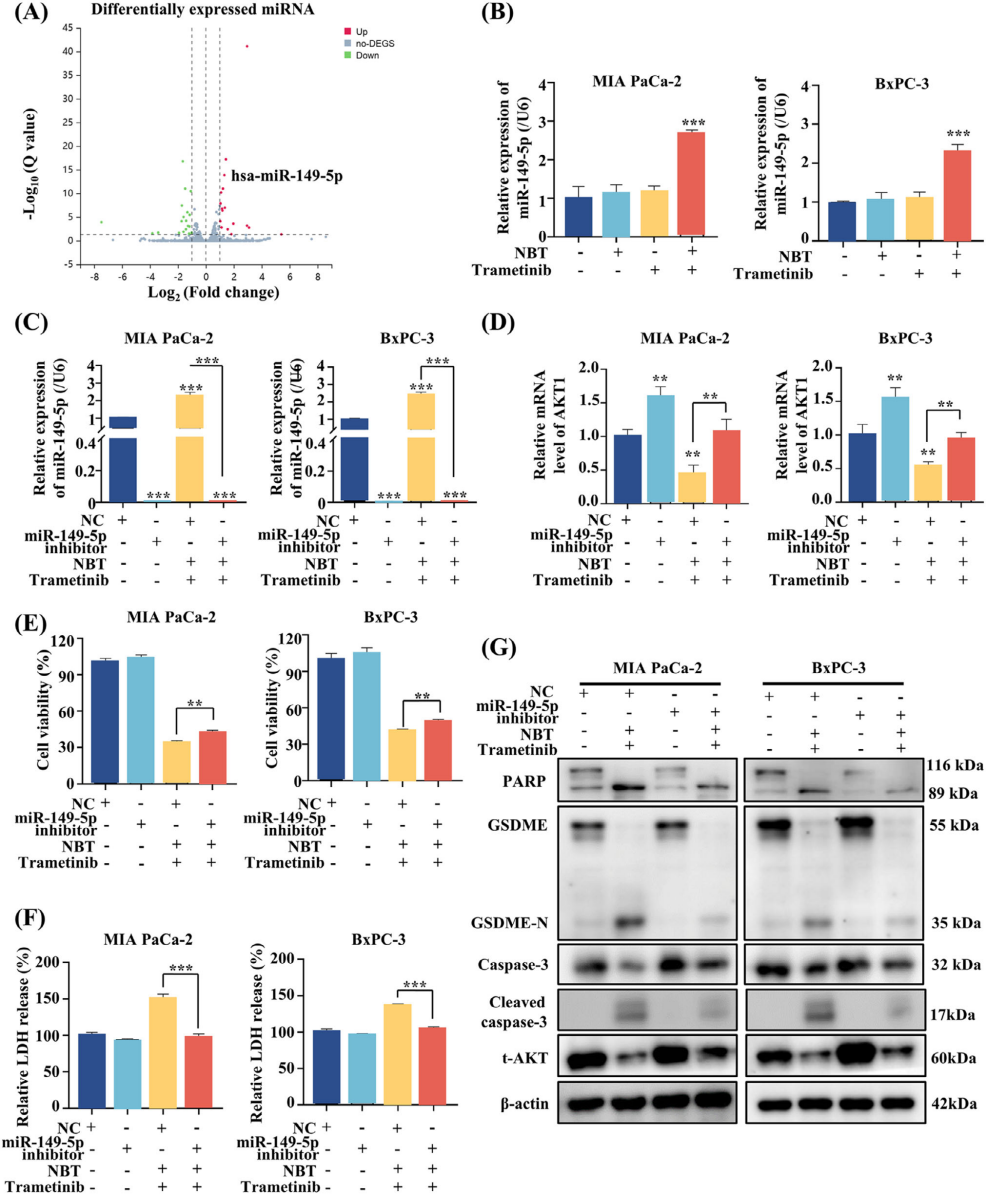
The combination of NBT and trametinib upregulated miR‐149‐5p expression. (A) The volcano plot of differentially expressed miRNA. (B) miR‐149‐5p expression after cotreatment with NBT and trametinib for 12 h was measured by RT‐PCR. (C) After transfection with miR‐149‐5p inhibitor or negative control for 48 h, miR‐149‐5p expression was measured by RT‐PCR. RNU6 was utilized as an internal control. (D) The mRNA levels of AKT1 were detected. 18S was utilized as an internal control. (E) The cell viability and (F) LDH release levels were determined. (G) The protein levels of GSMDE, PARP, caspase‐3 and t‐AKT were assessed by Western blot analysis. β‐actin was utilized as an internal control. Data are presented as the means ± SD from three independent experiments. **p < 0.01, ***p < 0.001.

To explore the upstream regulation of the AKT pathway induced by the combination of NBT and trametinib, microRNA (miRNA) sequencing analysis was conducted. Among the differentially expressed miRNAs, the increased expression of miR‐149‐5p emerged as a potential regulator, which was previously reported to target AKT (Figure 7A) [32]. RT‐PCR analysis further validated a significant upregulation of miR‐149‐5p after the cotreatment (Figure 7B). Subsequently, transfection of a miR‐149‐5p inhibitor into PDAC cells resulted in a marked reduction in miR‐149‐5p expression, subsequently upregulating AKT1 mRNA expression (Figure 7C,D). This effect partially attenuated the cell death and LDH release levels induced by the cotreatment (Figure 7E,F). Moreover, the downregulation of AKT protein induced by the combination treatment was significantly reversed upon administration of the miR‐149‐5p inhibitor. This reversal subsequently modulated the cleavage of GSDME and PARP, which had been induced by the combination of NBT and trametinib (Figure 7G). These findings suggested that the combination treatment may inhibit the AKT signaling pathway by upregulating miR‐149‐5p.

### 2.8 The Combination of NBT and Trametinib Inhibited Tumor Growth in Vivo

**FIGURE 8:**
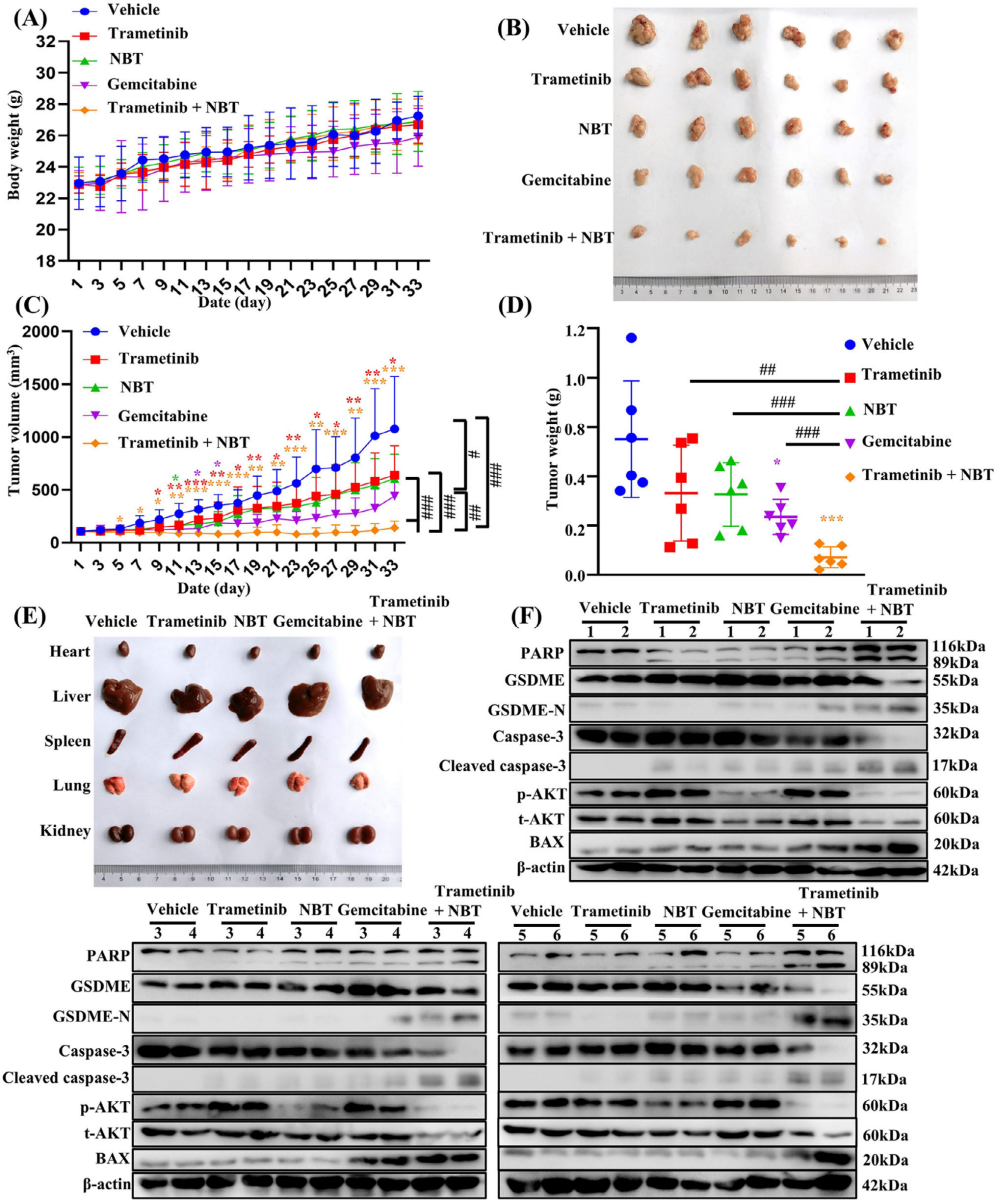
The combination of NBT and trametinib inhibited tumor growth in vivo. Xenograft mouse models were established with MIA PaCa‐2 cells and treated with vehicle, trametinib, NBT, gemcitabine, or the combination of NBT plus trametinib. Body weights (A) and tumor volume (C) were measured every 2 days. The images (B) and weights (D) were taken on the 33rd day. (E) Representative organ morphology of mice in each group. (F) Protein levels associated with apoptosis, pyroptosis and AKT signaling pathway were detected by Western blot, with β‐actin as the control. Data are presented as the means ± SD (*n* = 6). *p < 0.05, **p < 0.01, ***p < 0.001 versus vehicle; #*p* < 0.05, ##*p* < 0.01, ###*p* < 0.001 versus monotreatment.

To investigate the inhibitory effect of NBT and trametinib in vivo, MIA PaCa‐2 cells were subcutaneously injected into nude mice. The mice were treated with NBT at 2 mg/kg, trametinib at 0.4 mg/kg or a combination of both treatments every other day. Gemcitabine at 20 mg/kg was administrated twice a week as the positive control. No significant differences in body weights were observed between the vehicle group and the other treatment groups (Figure 8A). The cotreatment significantly suppressed tumor growth, resulting in smaller tumor volumes and weights compared with other groups (Figure 8B–D). Importantly, these effects were achieved without substantial organ toxicity in nude mice (Figure 8E).

The hematoxylin and eosin staining (H&E) results demonstrated a reduced tumor density in the treated groups compared with the control group, with the most significant reduction observed in the combination of trametinib and NBT. The IHC results revealed that tumor tissues in the combination treatment group exhibited higher expressions of cleaved caspase‐3 and lower expressions of p‐AKT compared with the vehicle and monotherapy groups (Figure S5). Western blot analysis was further performed to detect the protein expressions in tumor tissue. Consistent with the in vitro findings, the combination of NBT and trametinib prominently activated BAX and caspase‐3, leading to the cleavage of GSDME and PARP (Figure 8F). The combination treatment induced more pronounced apoptosis and pyroptosis compared with other treatments. Notably, that treatment with trametinib or gemcitabine resulted in an undesired increase in AKT phosphorylation levels, whereas the cotreatment significantly reversed this effect. These results confirmed that the combination of NBT and trametinib synergistically inhibited tumor growth in vivo by inducing pyroptosis and apoptosis, primarily through the downregulation of AKT.

## 3 Discussion

PDAC, a highly aggressive malignancy with a poor prognosis, urgently requires the development of novel therapeutic strategies. In recent years, with the increasing focus on pyroptosis, this form of immunogenic programmed cell death has emerged as a promising approach for the development of novel therapeutic strategies in PDAC [33]. Targeting this unique cell death mechanism of pyroptosis is possible to enhance antitumor immune responses and improve treatment outcomes [34, 35]. Continued research and clinical investigations are essential for translating the potential of pyroptosis into clinical practice, offering new hope for patients with PDAC. Although the study of pyroptosis in PDAC is still in its early stages, it holds great value and promises to advance treatment options.

Natural products have long been recognized as a valuable source of potential therapeutic agents for cancer treatment. Their complex chemical structures often interact with multiple targets and pathways involved in cancer progression. Combining natural products with conventional drugs holds great promise in cancer therapy, as it can lead to synergistic effects such as improved treatment efficacy, reduced toxicity, and overcoming drug resistance.

MEK, a crucial component of the rat sarcoma/rapidly accelerated fibrosarcoma/mitogen‐activated protein kinase kinase/extracellular signal‐regulated kinase (RAS/RAF/MEK/ERK) signaling cascade, has become a key target in cancer treatment. However, using MEK inhibitors alone results in reduced efficacy and drug resistance due to the complex crosstalk and compensation between MEK pathway and other signaling pathways, such as AKT and signal transducer and activator of transcription 3 (STAT3) pathways [11]. This highlights the need for combination therapies that can address these adaptive responses and improve clinical outcomes. Recent studies have demonstrated that MEK inhibitors could be effectively combined with multiple pathway inhibitors or natural products to counteract the aberrant activation of prosurvival pathways and enhance antitumor efficacy [36].

Our study revealed a compelling therapeutic effect for the treatment of PDAC both in vitro and in vivo with the combined use of NBT and trametinib. Notably, unlike many AKT inhibitors that primarily target AKT phosphorylation, the NBT and trametinib combination significantly inhibited AKT at the transcriptional level. This distinct mechanism may offer more durable suppression of tumor cell proliferation [37]. Consistent with previous findings, trametinib alone triggered AKT activation in PDAC cells, promoting survival and drug resistance [14]. However, when combined with NBT, AKT activation was effectively suppressed, leading to enhanced apoptosis and pyroptosis. Additionally, previous study has revealed that the inhibition of MEK signaling pathway may activate AKT phosphorylation through the crosstalk with the mTOR signaling pathway [27, 28]. Notably, our results showed that the ratio of phosphorylated proteins to their corresponding total proteins remained unchanged. This suggests that the decrease in phosphorylation resulted from a reduction in total protein levels, rather than direct inhibition of kinase activity. Additionally, we tested the combination in esophageal cancer KYSE150 cells and observed similar synergistic effects, further inducing apoptosis and pyroptosis via AKT pathway inhibition (Figure S6). These findings highlighted the wider application potential of utilizing NBT in combination with trametinib in cancers other than those with KRAS or BRAF mutations [38].

Moreover, the flow cytometry results showed that the combination of NBT and trametinib obviously elevated ROS levels, which suggested a synergistic effect in ROS production. This increase in ROS was crucial for inducing cell death, LDH release, and the cleavage of GSDME and PARP. Interestingly, neither NBT nor trametinib alone could increase the ROS level whereas the combination led to a significant increase. It has been demonstrated that the AKT pathway can regulate ROS levels, as AKT inhibitors have been shown to upregulate ROS levels [39]. In our study, the combination of trametinib and NBT significantly reduced both the phosphorylation and total protein levels of AKT, which may have further increased ROS levels. Further investigation revealed that the ROS inhibitor not only decreased AKT phosphorylation and protein levels but also impacted AKT transcriptional activity. Moreover, miRNA‐seq analysis showed that the combination treatment upregulated miR‐149‐5p, which is known to interact with AKT in cancer [32, 40, 41]. Our results demonstrated that the combination of NBT and trametinib upregulated the expression of miR‐149‐5p in PDAC cells, consequently downregulating the transcription of AKT1. The use of miR‐149‐5p inhibitor confirmed its negative regulation of AKT expression, thereby modulating pyroptosis and apoptosis. These findings revealed the potential role of miRNA‐149‐5p in regulating the AKT signaling pathway.

In conclusion, this study revealed that the combination of NBT and trametinib effectively induced pyroptosis and apoptosis by downregulating AKT signaling pathway (Figure S7). This novel combination therapy holds promise for improving treatment outcomes and overcoming drug resistance in PDAC patients. Furthermore, these findings may also provide valuable insights for the development of similar combination therapies in other types of cancer. By targeting the complex crosstalk of signaling pathways involved in tumor progression, this approach not only renews the therapeutic potential of trametinib but also highlights the innovative application of natural products in enhancing the clinical significance of contemporary cancer treatments. Despite the promising results, this study has certain limitations. First, due to the limited number of clinical samples, the significance of GSDME in pancreatic cancer treatment requires further evaluation. Second, the animal models used in this study have limitations; we utilized subcutaneous tumor models, which may not accurately reflect the clinical characteristics of pancreatic cancer. Patient‐derived xenograft models would likely provide a more suitable alternative. Third, while the study demonstrated that the combination induced GSDME‐mediated pyroptosis and apoptosis primarily through the PI3K/AKT pathway, it remains unclear whether these effects were direct or indirect. Furthermore, the synergistic mechanisms of NBT and trametinib were not fully elucidated. Consequently, its potential application in treating PDAC patients requires further validation for clinical use.

## 4 Materials and Methods

### 4.1 Reagents and Antibodies

NBT with a purity >98% was isolated from *Garcinia bracteata* in our lab as described in the previous study [20]. Details about the antibodies are provided in Table S1.

### 4.2 Patient Specimens

Tissue samples, comprising both normal and cancerous tissues, were collected from four PDAC patients (three males and one female) with confirmed histopathology, at Ruijin Hospital, Shanghai Jiaotong University School of Medicine (Table S2). None of the patients had undergone adjuvant chemotherapy or radiation treatment before surgery. This study was approved by the Ethics Committee of Ruijin Hospital, Shanghai Jiao Tong University School of Medicine, with the Ethical Review Approval Number (161) in 2021.

### 4.3 Immunohistochemistry (IHC) Analysis

Each specimen underwent standard histological processing, after which the tissue sections were stained by hematoxylin and eosin, incubated with GSDME, and cleaved caspase‐3 and p‐AKT antibodies.

### 4.4 Cell Lines and Cell Culture

Human PDAC cell lines MIA PaCa‐2 and BxPC‐3 were purchased from American Type Culture Collection. Both kinds of cells were cultured with RPMI‐1640 medium (Gibco, USA), supplemented with 10% fetal bovine serum (Gibco) and 1% penicillin‐streptomycin (Gibco), and incubated at 37°C in a humidified atmosphere with 5% CO_2_.

### 4.5 MTT Assay

The cell viability of PDAC cells after 24‐h treatment was assessed by MTT assay as described previously [21]. The synergistic effect of NBT and trametinib was analyzed using the CI [42, 43].

### 4.6 Colony Formation Assay

According to our previous study [21], MIA PaCa‐2 and BxPC‐3 cells with NBT (1 µM), trametinib (5 µM) or in a combination for 24 h were subjected to the colony formation assay.

### 4.7 Microscopy Imaging

PDAC cells were plated in a 6‐well plate and treated with NBT (1 µM) or trametinib (5 µM) or in a combination for 24 h. The bright‐field images of cells were captured by a Leica XSP‐8CA microscope to observe the cellular morphology after treatment. The pore‐forming activity of the cotreatment‐induced pyroptosis in PDAC cells was examined by TEM.

### 4.8 Cell Transfection

The siRNAs of GSDME and caspase‐3 were purchased from GenePharma (Shanghai, China). And miRNA inhibitor was purchased from BioTNT (Shanghai, China). Cells were plated in a six‐well plate and transfected with siRNAs or miRNA inhibitor using Lipofectamine 2000 (Invitrogen, Carlsbad, CA, USA) according to the manufacturer's instructions [44].

### 4.9 Gene Expression Correlation With Stage and Survival Analysis

The correlation between gene expression and patient pathological stage was analyzed using GEPIA (http://gepia.cancer‐pku.cn). The correlation between gene expression and OS and DFS was investigated using the Mantel–Cox test. The median expression level was utilized as the threshold to classify patients into high‐expression and low‐expression cohorts.

### 4.10 Sequencing of mRNA and miRNA

MIA PaCa‐2 cells were treated with NBT (1 µM), trametinib (5 µM), or in a combination for 12 h. Procedures for RNA extraction, library construction and sequencing on the BGISEQ‐500 platform have been described previously [45, 46]. The gene expression levels were estimated using the fragments per kilobase of exon per million fragments mapped. Differential expression analysis in each sample was performed using PossionDis with false discovery rate ≤0.05 and |Log2Ratio| ≥ 1. The enrichment analyses were conducted using the KEGG (http://www.genome.jp/kegg/). The raw RNA‐seq data have been deposited in the NCBI Gene Expression Omnibus under the accession number GSE275621.

### 4.11 Measurement of the ROS

The intracellular ROS levels of PDAC cells were detected by using a ROS detection kit according to the manufacturer's instructions. The fluorescence intensity was measured using a flow cytometer and analyzed by FlowJo software (v10.8.1).

### 4.12 Apoptosis Analysis

For apoptosis analysis, an Annexin V‐FITC apoptosis kit (BD Pharmingen, Franklin Lakes, NJ) was employed. PDAC cells were treated with NBT (1 µM), trametinib (5 µM), or in a combination for 24 h according to the manufacturer's instructions. The proportions of apoptotic and pyroptotic cells were determined using a flow cytometer (Becton & Dickinson Company, Franklin Lakes, NJ, USA), and the data were analyzed using FlowJo software.

### 4.13 Real‐Time PCR (RT‐PCR)

PDAC cells were subjected to NBT (1 µM), trametinib (5 µM), or in a combination for 12 h. Total RNA of cells or PDAC patient tissues were extracted using TRIzol (TaKaRa, Kusatsu, Shiga, Japan) followed by synthesis of cDNA via reverse transcriptase (TaKaRa). The expression of miRNA was detected by miRNA RT‐PCR kit (BioTNT) following the manufacturer's instructions. Quantitative analysis was performed as described previously [44]. The primers used for RT‐PCR were present in Table S3.

### 4.14 Western Blot

The expressions of proteins of PDAC cells after treatment and tumor tissues were detected by Western blot as described previously [44].

### 4.15 Xenograft Tumor Model

All animal experiments were approved by the University's Animal Care and Use Committee (Ethical Committee Number: PZSHUTCM220725007). Four‐week‐old male BALB/c‐nude mice were purchased from Shanghai Sippr‐BK Laboratory Animal Co. Ltd. (Shanghai, China). Each mouse was then subcutaneously injected with 2 × 10^6^ MIA PaCa‐2 cells into the right back. After tumors reached 100 mm^3^, mice were divided into five groups (*n* = 6) and treated with NBT (2 mg/kg), trametinib (0.4 mg/kg), or their combination every other day. The vehicle group received a solvent control, and gemcitabine (20 mg/kg) was used as a positive control. Tumor volume was calculated daily. On day 33, the mice were sacrificed and the tumors and organs were collected for further analysis.

### 4.16 Statistical Analysis

All data are presented as means ± SD from three independent experiments. The comparisons between the two groups were made by Student's *t*‐test; comparisons among multiple groups were made by one‐way or two‐way ANOVA with Tukey's post hoc tests. GraphPad Prism 8.0.2 software (GraphPad, San Diego, CA, USA) was utilized for statistical analysis and visualization. Values of *p* < 0.05 were considered statistically significant.

## Author Contributions

J.Q.T. performed experiments and wrote the paper. Z.Y.B., K.Q., and L.J.Z. performed experiments. C.W.Z. analyzed the data. J.B.J. and L.Z. designed experiments and revised the manuscript. H.X.X. supervised the study and revised the manuscript. All authors have read and approved the final manuscript.

## Ethics Statement

The patient sample‐related experiments were approved by the Ethics Committee of Ruijin Hospital, Shanghai Jiao Tong University School of Medicine, with the Ethical Review Approval Number (161) in 2021 and compliant with all relevant ethical regulations. Written informed consent for research purposes was obtained from the patients. All animal studies were approved by the Animal Care and Use Committee of the University Institution (approval number PZSHUTCM 220725007).

## Conflicts of Interest

The authors declare no conflicts of interest.

## Supporting information



Supporting Information

## Data Availability

The data are available from the corresponding author on reasonable request.
