# Supplementary material for: Neobractatin and Trametinib Synergistically Induce Apoptosis and Gasdermin E‐Dependent Pyroptosis in Pancreatic Cancer Cells
Source: MedComm (2020). 2025 Jul 1;6(7):e70250. doi: 10.1002/mco2.70250 (PMC12209591; doi:10.1002/mco2.70250)
Supplement: Supplementary file 1 — Supporting Information [file MCO2-6-e70250-s001.pdf]

Neobractatin and trametinib synergistically induce apoptosis and gasdermin E - dependent pyroptosis in pancreatic cancer cells

Jiaqi Tan [1,#], Ziyi Bao [1,#], Kai Qin [2,3], Liujing Zhu [1], Changwu Zheng [1], Jiabin Jin [2,3*], Li Zhang [1,*], Hongxi Xu [4,*]

[1] School of Pharmacy, Shanghai University of Traditional Chinese Medicine, Shanghai, 201203, China

[2] Department of General Surgery, Pancreatic Disease Center, Ruijin Hospital, Shanghai Jiao Tong University School of Medicine, Shanghai, China.

[3] Research Institute of Pancreatic Diseases, Shanghai Jiao Tong University School of Medicine, Shanghai, China

[4] Shuguang Hospital, Shanghai University of Traditional Chinese Medicine, Shanghai 201203, China

[*]Correspondence

Jiabin Jin, Department of General Surgery, Pancreatic Disease Center, Ruijin Hospital, Shanghai Jiao Tong University School of Medicine, Shanghai, China.

Email: jjb11501@rjh.com.cn

Li Zhang, School of Pharmacy, Shanghai University of Traditional Chinese Medicine, Shanghai, 201203, China.

Email: zhangli1003ecpu@126.com

Hongxi Xu, Shuguang Hospital, Shanghai University of Traditional Chinese Medicine, Shanghai 201203, China.

Email: xuhongxi88@gmail.com

[#]Jiaqi Tan and Ziyi Bao contributed equally to this work.

Table S1: List of antibodies used in this study

Antibody	Company name	Catalogue number	Dilution
GSDME	Abcam	Ab215191	1:1000
IKKα	Abcam	Ab32041	1:1000
mTOR	Abcam	Ab134903	1:1000
AKT	Cell Signaling Technology	9272	1:1000
ERK	Cell Signaling Technology	4695	1:1000
MEK	Cell Signaling Technology	9122	1:1000
Rictor	Cell Signaling Technology	2114	1:1000
p-AKT	Cell Signaling Technology	4060	1:1000
p-IKKα/β	Cell Signaling Technology	2697S	1:1000
p-mTOR	Cell Signaling Technology	5536S	1:1000
p-MEK	Cell Signaling Technology	9154	1:1000
p-ERK	Cell Signaling Technology	4370	1:1000
p-Rictor	Cell Signaling Technology	3806	1:1000
Caspase-3	Cell Signaling Technology	9665	1:1000
Caspase-9	Cell Signaling Technology	9502	1:1000
PARP	Cell Signaling Technology	9542	1:1000
β-actin	Proteintech	66009-1-lg	1:10000
BAX	Proteintech	50599-2-lg	1:1000

Table S2: Patient information for pancreatic cancer tissue

Patient ID	Age	Gender	Case Number	Cancer Subtype	Histology	Tumor Characteristics	Margin Involvement	Lymph Node Involvement	Other Involvement	Other
z849087	48	M	2020-60806	Pancreatic head ductal adenocarcinoma	Medium-low differentiation	Volume: 3.5×3.0×2.5 cm, tumor invasion into peripancreatic fat tissue, nerve invasion present, no intravascular tumor thrombus	Pancreatic neck margin involved with cancer tissue	Bile duct margin clear of cancer tissue, gastric margin clear of tumor involvement, duodenal margin clear of tumor involvement	Nearby lymph nodes: 1 out of 4 lymph nodes around the pancreas shows cancer infiltration/metastasis	Chronic cholecystitis
z847290	81	M	2020-58657	Pancreatic tail ductal adenocarcinoma	Moderate differentiation	Volume: 3.5×1.5×1.5 cm, tumor invasion into peripancreatic fat tissue, nerve invasion present, intravascular tumor thrombus present	Pancreatic margins are clear of cancer tissue	Nearby lymph nodes: 7 out of 14 lymph nodes around the pancreas show cancer metastasis	Spleen - fibrofatty tissue at the splenic hilum involved, splenic parenchyma and greater omentum - no cancer observed	N/A

Continue

Patient ID	Age	Gender	Case Number	Cancer Subtype	Histology	Tumor Characteristics	Margin Involvement	Lymph Node Involvement	Other Involvement	Other
z847500	70	M	2020-58516	Pancreatic tail ductal adenocarcinoma	Moderate differentiation	Volume: 6.7×2.7×2.0 cm, tumor invasion into peripancreatic fat tissue, nerve invasion present, no intravascular tumor	Pancreatic margins are clear of cancer tissue	Nearby lymph nodes: 3 out of 6 lymph nodes adjacent to the tumor show cancer metastasis	Spleen - no cancer tissue observed	N/A
z848662	79	F	2020-61776	Pancreatic head ductal adenocarcinoma	Medium-low differentiation	Volume: 2.0×1.5×1.0 cm, tumor invasion into peripancreatic fat tissue, nerve nnvasion present, no intravascular tumor thrombus	Bile duct margin - no tumor involvement, peri-gastric margin - no tumor involvement, duodenal margin - no tumor involvement	Pancreatic peri-lymph node metastasis - none	Spleen - no significant lesions, stomach - a nodule (consistent with GIST, diameter 0.3 cm), small intestine segment - no cancer observed, greater omentum - no cancer observed	Serosal nodule CD34 (+), CD117 (+), DOG-1 (+), Desmin (partially +), SMA (partially +), S100 (-), SDHB (protein expression), Ki67 (approximately 5% +)

Table S3. Primer sequences used in this study

Gene	Sequence
AKT1-F	5' CTTCAAGCCCCAGGTCAC 3'
AKT1-R	5' CGCTGTCCACACACTCCAT 3'
GSDME-F	5' GAGCCAGTCTTCATTTGGAAC 3'
GSDME-R	5' GAGCACAGGGTTTCTCAGATT 3'
18S-F	5′ GTAACCCGTTGAACCCCATT 3′
18S-R	5′ CCATCCAATCGGTAGTAGCG 3′
Has-miR-149-5p-F	5′ GTCTGGCTCCGTGTCTT 3′
Has-miR-149-5p-R	5′ AACTGGTGTCGTGGAGTC 3′
RUN6-F	5′ GCTTCGGCAGCACATAT 3′
RUN6-R	5′ ATTTGCGTGTCATCCTTG 3′

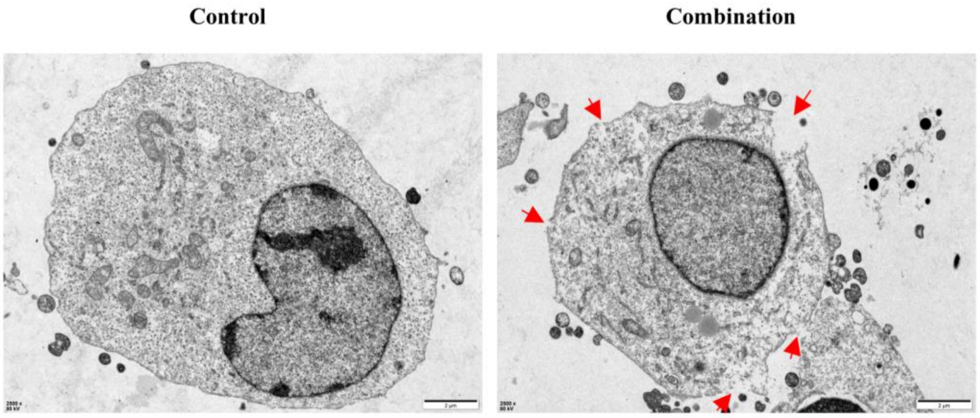

Figure S1. TEM images of MIA PaCa-2 cells after cotreatment of NBT and trametinib for 24 h. The red arrowheads indicate the emerging pore from the plasma membrane.

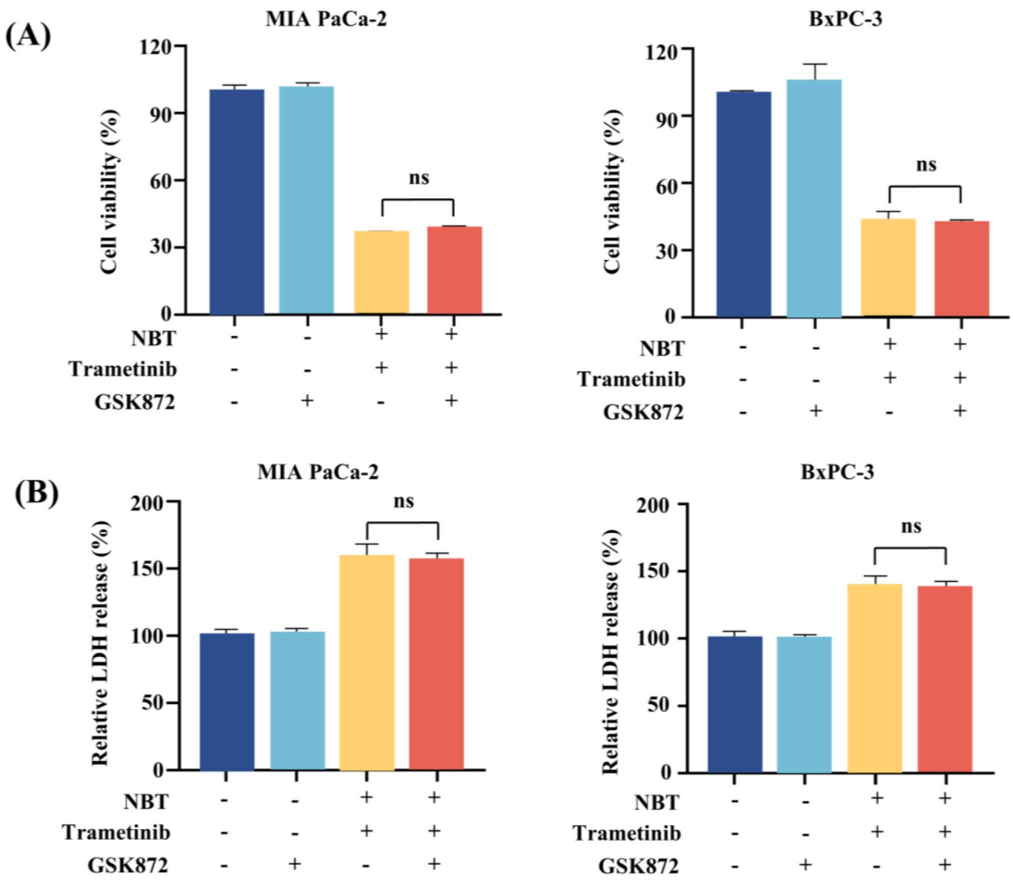

Figure S2. The combination of NBT and trametinib induced pyroptosis rather than necroptosis. (A) The cell viability and (B) LDH release level were detected with or without GSK872 (2 μM). Data are presented as the means ± SD from three independent experiments.

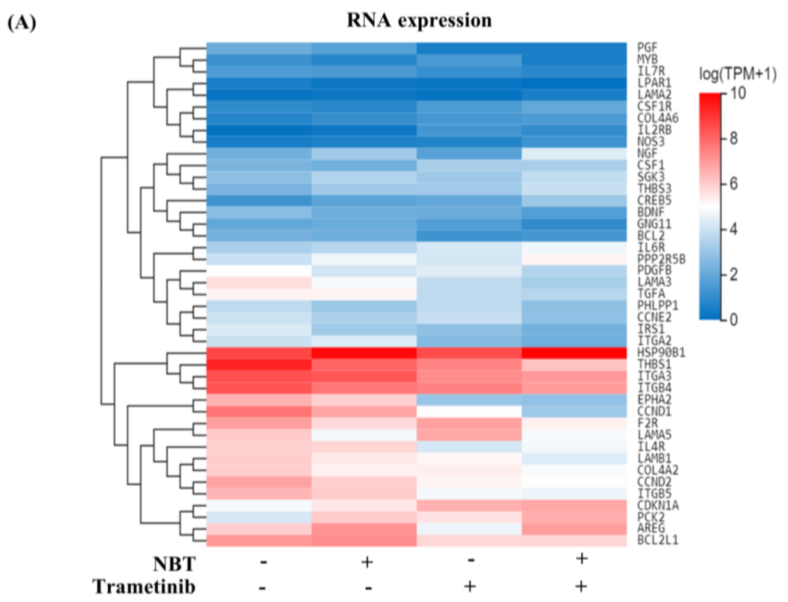

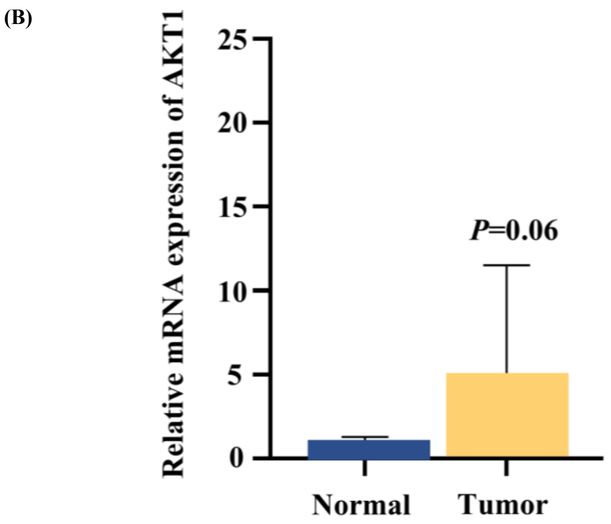

Figure S3. AKT plays an important role in the combination of NBT and trametinib in PDAC. (A) A heatmap was drawn to depict the expression levels of differentially expressed genes involved in the PI3K/AKT signaling pathway. (B) The mRNA level of AKT1 in PDAC tissues and adjacent normal tissues from 4 patients. 18S was utilized as an internal control. Data are presented as the means ± SD from three independent experiments.

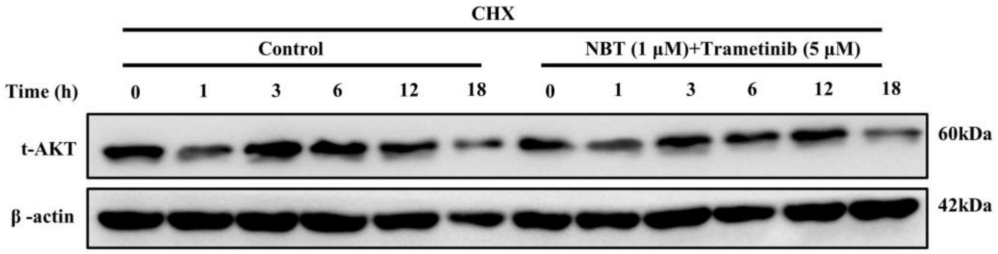

Figure S4. The degradation of AKT protein was not affected by the treatment of NBT and trametinib. MIA PaCa-2 cells were treated with CHX (40 μg/mL), the protein level of AKT was analyzed by Western blot. β-actin was utilized as an internal control.

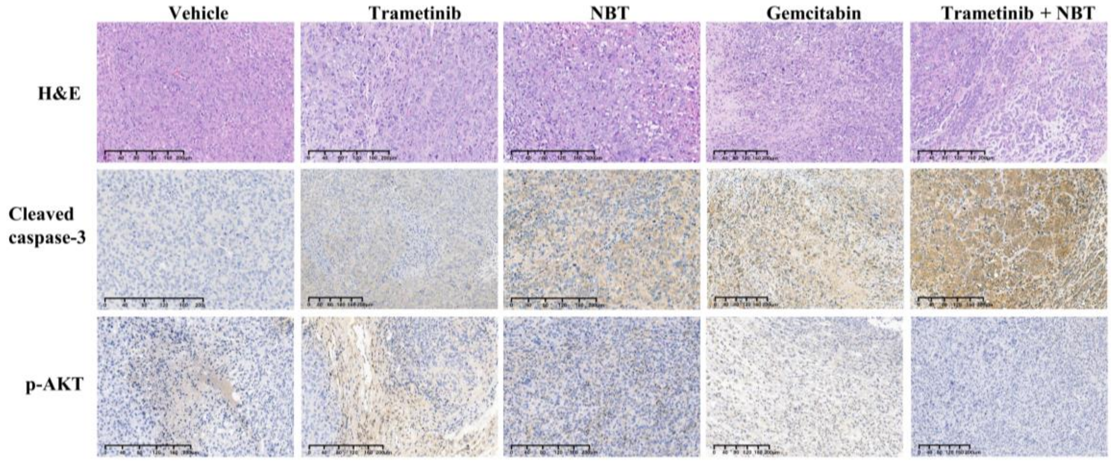

Figure S5. Representative images of H&E staining and immunohistochemical staining for cleaved caspase-3 and p-AKT in tumor sections treated with the vehicle, trametinib, NBT, gemcitabine and combination. Scale bar=200 μm.

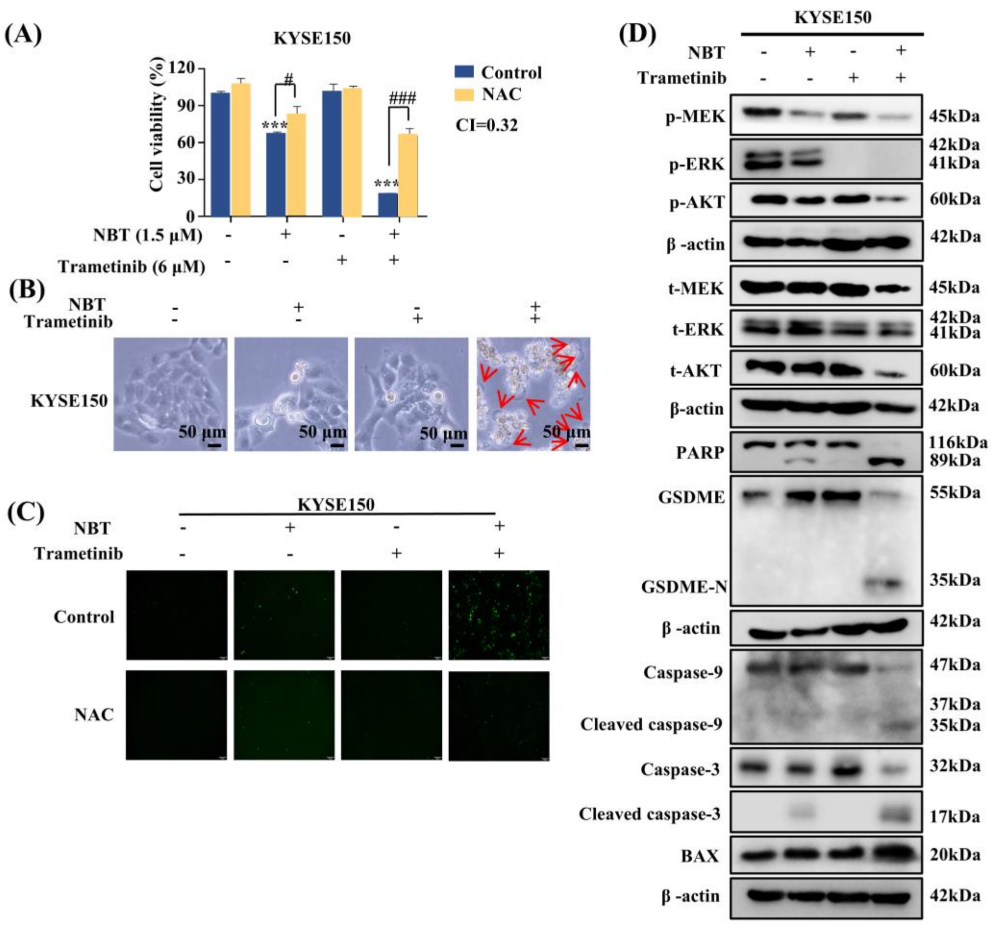

Figure S6. The combination of NBT and trametinib synergistically demonstrated an inhibitory effect on KYSE150 cells through the AKT pathway. (A) The cell viability of KYSE150 cells was detected by MTT assay and the CI values were calculated. (B) Bright-field images were taken after treatment to observe the cellular morphology (large bubbles are indicated by red arrows). (C) The fluorescence of ROS level was detected after treatment with or without NAC. (D) The relative protein levels were analyzed by Western blot. β-actin was utilized as an internal control. Data are presented as the means ± SD from three independent experiments. ***P < 0.001 versus control; #P < 0.05, ###P < 0.001 versus monotreatment.

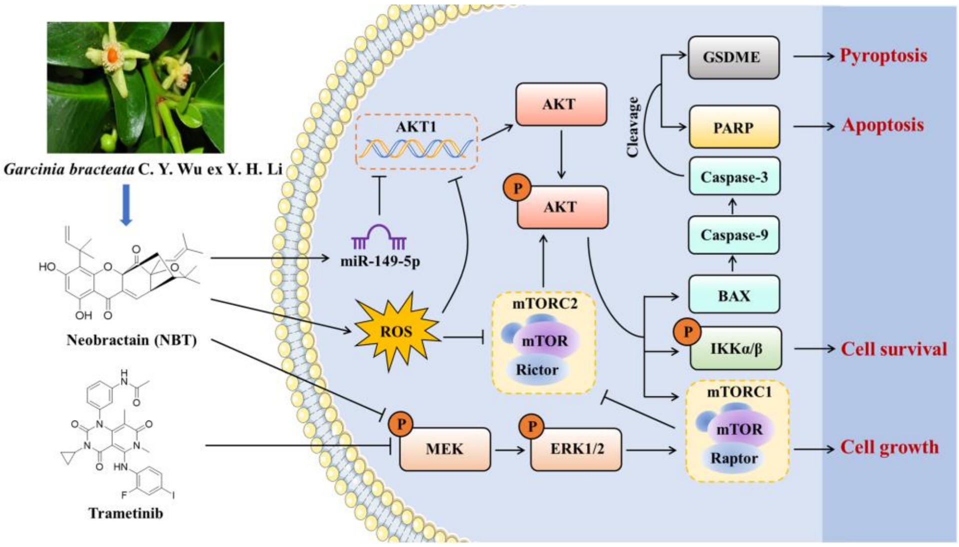

Figure S7. Neobractatin, a natural compound derived from Garcinia bracteata, significantly inhibited the growth of PDAC cells when used in combination with trametinib. This combination therapy promoted cell death through GSDME-mediated pyroptosis and apoptosis. Mechanistically, the combined treatment elevated levels of ROS, which in turn suppressed the AKT signaling pathway and its downstream targets, thereby enhancing the anti-tumor efficacy against PDAC cells.
